# Comparison of Colposcopic Biopsy Results of Patients Who have Cytomorphological Normal but HPV 16-18 or Other High-Risk HPV Subtypes Positive

**DOI:** 10.31557/APJCP.2019.20.2.417

**Published:** 2019

**Authors:** Hüseyin Aydoğmuş, Serpil Aydoğmuş

**Affiliations:** 1 *Atatürk Research and Training Hospital, *; 2 *School of Medicine, Department of Gynecology and Obstetrics, İzmir Katip Çelebi University, Turkey. *

**Keywords:** Cervical cytology, colposcopy, high risk human papilloma virus subtypes

## Abstract

**Objective:**

Current guidelines suggest annual co-test follow-up in non-16/18 high-risk HPV positive patients without abnormal cytologic findings. Considering the relatively high false-negative rates of cervical cytology, a follow-up period of one year might constitute an additional risk for development of cervical malignancies in these patients. The current study aims to evaluate colposcopic biopsy results of cytologically normal patients detected to carry high risk HPV by screening tests.

**Materials and Methods:**

A total of 160 patients with normal cervical cytology and high-risk HPV subtypes who underwent colposcopic examination were included in the study. Patients were divided into two groups according to their HPV results: HPV 16-18 and other types (non-16/18 high-risk subtypes). ECC, cervical biopsy, LEEP/conization results were analyzed for both groups. Histopathological results of the groups were compared in terms of ≤LSIL, HSIL and cervical cancer rates.

**Results:**

Cervical biopsy results in the “16-18” group were assessed as HSIL in 40 (48.2%) patients, LSIL in 6 patients (7.2%) and normal in 37 (44.6%) patients. ECC results revealed HSIL in 9 (10.8%) patients and LSIL in 1 (1.2%) patient. Nineteen out of 42 patients who underwent LEEP/conization had HSIL (surgical margin positivity was reported in 4 cases), while 3 patients had LSIL. None of the cases had cervical carcinoma.

**Conclusion:**

The present study detected that 15.6% of women infected with non-16/18 high-risk HPV subgroups developed ≥HSIL lesions. Although this rate seems lower than HPV 16-18 group, it is still too high to be overlooked. In conclusion, we suggest further clinical trials with larger number of patients to be conducted on this topic.

## Introduction

National Cancer Institute, Centers for Disease Control and Prevention, Jemal et al., (2013) and Viens et al., (2016) report that HPV infection is associated with nearly all (more than 90%) of cervical cancers (National Cancer Institute, 2011; Centers for Disease Control and Prevention, 2012). HPV 16 alone is responsible from half of all cervical cancers, while HPV 18 is responsible from 20% (National Cancer Institute, 2011). Fifteen HPV subtypes (HPV 16, 18, 31, 33, 35, 39, 45, 51, 52, 56, 58, 59, 68, 73, 82) are defined as high-risk (hrHPV) for the development of cervical cancer by Centers for Disease Control and Prevention, 2011; Jemal et al., 2013)

The ASCCP guidelines recommended immediate colposcopy in HPV 16/18 positive cases, regardless of cytology results, while co-testing after 1 year is suggested in the presence of other hrHPV subtypes with a negative cytology (ASCCP Guidelines, 2013). Two studies conducted in Turkey reported 20% of women with normal cervical cytology to be HPV-DNA positive and 32.5% of hrHPV positive women to have normal cervical cytology (Dursun at al., 2009; Beyazit at al., 2018). Various studies have reported the false negative rate of cervical cytology to be between 15% and 63% (Lönnberg at al., 2010; Banna at al., 2014). Additionally, patients might interrupt their annual follow-ups for various reasons. 

The aim of this study is to compare colposcopic biopsy results of HPV 16/18 positive patients and patients carrying at least one of the hrHPV subtypes both with normal cervical cytology.

## Materials and Methods

This retrospective cohort study has been conducted between January 2015 and December 2017 in a tertiary center located in western Turkey at the Clinic of Obstetrics and Gynecology, Izmir Katip Çelebi University Ataturk Training and Research Hospital. A total of 167 women referred to our hospital due to HPV DNA positivity from primary screening centers were included in the study. The study protocol was approved by the İzmir Katip Çelebi University Ethics Committee (2017-308). Patients who did not accept colposcopy and biopsy, or had previous history of cervical dysplasia were excluded from the study. Informed consent was obtained from all participants and the study was in agreement with the Declaration of Helsinki for Medical Research Involving Human Subjects. Cervical cytologic evaluation was performed by conventional or liquid-based cytology. Abnormal histopathologic findings were classified as low-grade squamous intraepithelial lesion (LSIL), high-grade squamous intraepithelial lesion (HSIL), and squamous cell carcinoma (SCC). Hybrid Capture2 (Qiagen) kit for HPV-DNA scans and CLART kit (Genomica) or Cobas^®^ HPV kit (Roche) for HPV genotyping were utilized. A total of 160 patients who were examined for at least one of the high-risk DNA subtypes and were found to have no abnormal findings on cervical cytology were included in the final statistical evaluation. All patients were evaluated by a gynecologist experienced in colposcopy (HA). Patients were divided into two groups according to HPV results as HPV 16/18 positive (group 1, HPV 16/18) and as non-16/18 hrHPV subtypes (group II, OTHERS). Endocervical curettage (ECC), cervical biopsy, LEEP/conization results of both groups were analyzed. Histopathologic results of the groups were compared in terms of ≤LSIL, HSIL and cervical squamous cancer rates. Eighty-three patients in the HPV 16/18 group and 77 patients in the ‘OTHERS’ group were evaluated.

**Figure 1:**
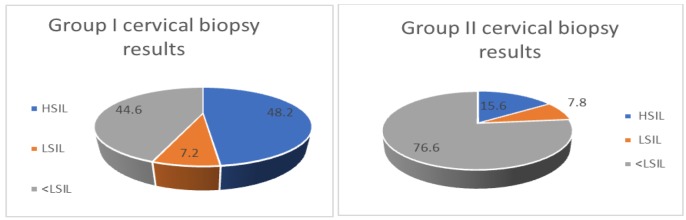
Cervical Biopsy Results of Groups

**Table 1:** Demographic Features of the Patients

	Group I HPV 16-18 (n=83)	Group II OTHERS (n=77)	p
Age (years)	44 (28-66)	45 (30-66)	0.280
Menopausal status (premenopausal) (n, %)	60 72.3	52 68.8	0.726
Parity	2 (0-6)	2 (0-5)	0.729

**Table 2:** Evaluation of Histopathological Findings

	Cervical Biopsy			ECC			LEEP		
	Group I	Group II		Gorup I	Group II		Gorup I	Group II	
	HPV 16-18	OTHERS	p	HPV 16-18	OTHERS	p	HPV 16-18	OTHERS	P
	n %	n %		n %	n %		n %	n %	
HSIL	40 48.2	12 15.6		9 10.8	1 1.3		19 45.2	8 66.7	
LSIL	6 7.2	6 7.8	<0.001	1 1.2	1 1.3	0.025	3 7.2	0 0	<0.001
< LSIL	37 44.6	59 76.6		73 88	75 97.4		20 47.6	4 33.3	
Total	83 100	77 100		83 100	77 100		42 100	12 100	

## Results

The median age of the “16-18” and the “others” group was 44 (28-66) and 45 (30-66), respectively. While 72.3% of the patients were premenopausal and 27.7% were postmenopausal in the “16-18” group, 68.8% were premenopausal and 31.2% were postmenopausal in the “others” group. Median parity of the both groups was 2 (0-6 in the “16-18” group and 0-5 in the “others” group) (Table 1). 

Cervical biopsy results in the “16-18” group were assessed as HSIL in 40 (48.2%) patients, LSIL in 6 patients (7.2%) and normal in 37 (44.6%) patients. ECC results revealed HSIL in 9 (10.8%) patients and LSIL in 1 (1.2%) patient. Nineteen out of 42 patients who underwent LEEP/conization had HSIL (surgical margin positivity was reported in 4 cases), while 3 patients had LSIL. None of the cases had cervical carcinoma.

The most common subtype in the “Others” group was found to be HPV 52 (15.5%). Cervical biopsy results were assessed as HSIL in 12 (15.6%) patients, LSIL in 6 (7.8%) patients and normal in 59 (76.6%) patients. ECC results revealed 1 HSIL (1.3%) and 1 LSIL. Eight out of 12 patients who underwent LEEP/conization had HSIL (surgical margin positivity was reported in a single case), while none had LSIL nor cervical carcinoma.

Evaluation of the groups regarding histopathological findings showed that 48.2% of the cases in the “16-18” group had ≥HSIL lesions while only 15.6% of the “others” group had ≥HSIL lesions (p˂0.01). Presence of HPV 16-18 resulted in 5-fold increased risk of developing ≥ HSIL lesions compared to other high-risk HPV subtypes (OR: 5.03% 95% CI 2.3-10.6).

## Discussion

We found that the detection rate of ≥HSIL lesions was significantly higher in HPV 16/18 positive cases compared to other hrHPV subtypes (48.2% vs 15.6%, p <0.001). The most frequent subtype except HPV 16/18 was HPV 52. 

Current guidelines recommend annual co-test follow-up in non-16/18 hrHPV infected women with negative pap-smears, since the risk of cervical preinvasive lesion or cancer development is directly proportional with persisting HPV infection and passing time (ASCCP Guidelines, 2013). Delayed diagnosis might lead to patients encountering severe epithelial changes (Cobos at al., 2014). However, it has been reported that false negative rate of pap-smear test is about 15–65% (Castillo at al., 2016). In this case, non-invasive follow-up of non-16/18 hrHPV cases for 1 year should be questioned due to the probability of increased risk for cervical dysplasia. 

A study investigating detectability of biopsy-confirmed high-grade cervical lesions by pap-smear reported that false low-grade cytomorphology rates were higher in non-16/18 hrHPV and mixed hrHPV infected women than HPV 16/18 infected women. In light of this the authors suggested that diagnosis-treatment approach might be delayed due to increased pap test underdiagnosis rates (Samimi at al., 2018). Another problem is the lack of annual co-test follow-up compliance by patients. In a study nearly half of the patients were lost to follow-up (Thrall at al., 2010). 

Despite the widespread incidence of hrHPV infections especially among young females, majority of infections are transient and spontaneously eliminated (NIH: Cervical Cancer Screening guideline Updated, 2018). Moreover, slow progressive nature of the infection prolongs the development time of an invasive lesion, thereby increasing chances of recognition in the precancerous stage (Ghosh et al., 2014). A previous study reported that detection rates of CIN3+ cervical lesions by primary HPV screening were 50% higher than scanning by cytology, however the number of colposcopies were doubled simultaneously (Wright at al., 2012). 

In our study, comparison of initially negative cytology HPV 16/18 positive patients with non-16/18 hrHPV positive patients regarding development of ≥ HSIL lesions yielded a 5-fold increased risk in the HPV 16/18 group. Although that risk is high enough to be ignored, given the slow natural course of the disease, potentially treatable preinvasive lesions, and increased demand for personnel and equipment due to colposcopic examination of cytology negative hrHPV positive patients, recommending annual co-test follow-up to smear negative non-16/18 hrHPV positive cases sounds reasonable. 

In conclusion, the risk of cervical preinvasive lesions among hrHPV infected cases without cervical cytomorphological abnormalities is 5-fold higher in HPV 16/18 positive cases. Although follow-up at one year is theoretically risky in non-16/18 hrHPV positive but cytology negative women, annual follow-up is a reasonable approach due to slow natural course of the disease. 

## Conflict of interest

Authors declare no conflict of interest or any funding.
